# Nanolignin and Nanochitin as Skin-Friendly Biopolymer Carriers for Innovative Cosme-Nutraceuticals: A Review

**DOI:** 10.3390/polym18212703

**Published:** 2026-11-01

**Authors:** Pierfrancesco Morganti, Maria-Beatrice Coltelli, Stefano Scialla

**Affiliations:** 1 R&D Center, Nanotechnology Unit, Academy of History of Healthcare Art, 00193 Rome, Italy; maria.beatrice.coltelli@unipi.it (M.-B.C.); stefano.scialla@unicampus.it (S.S.); 2 Dermatology Department, The First Hospital, China Medical University, Shenyang 110001, China; 3 Department of Civil and Industrial Engineering, University of Pisa, 56122 Pisa, Italy; 4 Department of Engineering, Università Campus Bio-Medico di Roma, 00128 Rome, Italy

**Keywords:** nanolignin, chitin nanofibrils, polyelectrolyte complexes, tissue-carrier, cosmeceutical, nutraceutical, waste biomass, circular economy

## Abstract

Lignin and chitin are abundant structural biopolymers that can be recovered from wood-processing, agricultural, fishery and other biological residues. Their conversion into nanoparticles, nanocrystals or nanofibrils provides opportunities to combine carrier functions with properties intrinsic to the polymers. This review examines the chemical structures, biological organization, isolation routes and nanostructuring methods of lignin and chitin, and relates these features to their potential use in cosmetic and nutraceutical delivery systems. Particular attention is given to the influence of raw material source and processing on particle morphology, surface chemistry, colloidal stability and functional performance. The UV-screening and antioxidant properties of nanolignin are discussed alongside the structural and surface-charge characteristics of nanochitin. Polyelectrolyte complexation is considered as an assembly strategy within this broader framework, with examples based on either polymer or chitin nanofibril–nanolignin complexes. Electrospun non-woven carriers and related delivery formats illustrate how these materials can be incorporated into functional products. Finally, the review identifies the characterization, safety, regulatory, manufacturing and environmental challenges that must be addressed before these very promising materials can be widely used in cosmetic and nutraceutical applications.

## 1. Introduction

The progressive depletion of fossil resources, together with the accumulation of non-degradable waste on lands and in the oceans, has made the replacement of petroleum-derived ingredients one of the priorities of the cosmetic and nutraceutical industries [1,2,3,4]. In this context, the biomass discarded by agriculture, forestry, fishery and the food industry should not be regarded as a disposal problem, but as an underexploited reservoir of polymers that are naturally biodegradable, skin-compatible and, in many cases, biologically active. Lignin and chitin, the two most abundant natural polymers on Earth after cellulose, may be obtained in large quantities and at low cost from wood-processing and crustacean waste, respectively [5,6,7], thus safeguarding the precious natural raw materials of the Earth while giving value to materials that would otherwise be burnt or landfilled [8]. Biodegradability provides an additional rationale for using these polymers as alternatives to persistent carrier materials. Chitin can undergo enzymatic hydrolysis to oligosaccharides and N-acetyl-D-glucosamine [9], whereas lignin can be biodegraded by specialized microorganisms through oxidative pathways [10].

A second driver is specific to the cosmetic sector. The current production of cosmetics is based on emulsions, with a large consumption of water and energy [11,12,13]. It has been calculated that, for the approximately 120 billion cosmetic products manufactured annually, a consumption of between 50 and 80 litres of water per kilogram would be necessary, bearing in mind that manufacturing requires 60% water for creams and 80–90% for shampoos and solutions [11,12]. Emulsions moreover require preservatives, emulsifiers, fragrances and colors that are frequently responsible for allergic and sensitizing phenomena [14,15], and are generally packed in plastic containers whose disposal contributes to the pollution problem the industry wants to address.

Lignin- and chitin-based carriers offer opportunities to address some of these challenges through alternative delivery formats. For example, non-woven tissues incorporating chitin–lignin complexes can carry active ingredients in a solid format rather than in a water-rich emulsion [16,17,18,19]. Such formats can reduce the water content of the finished product, allow formulations without emulsifiers, and permit packaging in materials such as paper or aluminium foil rather than conventional plastic containers [11,12].

For these carrier applications, processing lignin and chitin into nanoscale forms increases the interfacial area available for interactions with active ingredients and other formulation components. Colloidal lignin particles provide surfaces for cargo association and coating, while nanochitin, including nanofibrils and nanocrystals, offers different morphologies for interfacial stabilization and the construction of composite delivery matrices [20,21,22,23,24,25,26]. Accessible surface groups also support aqueous complexation between appropriately charged components, enabling active ingredients to be incorporated during assembly [27,28]. These nanoscale building blocks therefore offer opportunities to tailor cargo retention and release through carrier composition and architecture, whether they are used in dispersed systems or incorporated into larger assemblies and solid matrices. Improved delivery, however, also depends on the formulation and is not an automatic consequence of nanoscale size alone [24,25,26,29,30,31].

The present review considers nanolignin and nanochitin as functional carrier materials for cosmetic and nutraceutical applications, both separately and in combination. These applications encompass topical cosmetic products and orally administered nutraceutical products intended to support skin health and appearance, collectively referred to here by the umbrella term “cosme-nutraceuticals”. The review first examines how polymer structure, biological source and isolation determine the properties of the nanoscale materials. It then considers non-covalent assembly and carrier design, including polyelectrolyte complexes, protein-coated particles, Pickering emulsions and non-woven delivery systems. In this context, chitin nanofibril–nanolignin complexes, in which the two polymers are used together in nanostructured form, are discussed as a prominent example.

Lignin valorisation as a fuel or as a source of bulk aromatic chemicals, the chemistry of chitosan as a soluble polycation, and the extensive literature on lignin-filled composites for structural applications are outside the scope of this review and are only mentioned where they bear on carrier performance.

The review is organized as follows. Section 2 and Section 3 summarize the structure, biosynthesis, isolation and nanostructuring of lignin and chitin, respectively, together with the functional properties relevant to their use as carriers. Section 4 sets out the physicochemical basis of non-covalent complexation between oppositely charged nanopolymers, surveys published carrier systems based on lignin nanoparticles and chitin nanofibrils, examines chitin nanofibril–nanolignin complexes within this framework, and discusses delivery formats derived from these complexes. Section 5 and Section 6 address safety/regulatory considerations and product quality, respectively. Finally, Section 7 presents the main conclusions and potential directions for future research.

## 2. Lignin: Structure, Isolation, Nanoscale Production and Functional Properties

### 2.1. Structure and Biological Function

**Figure 1:**
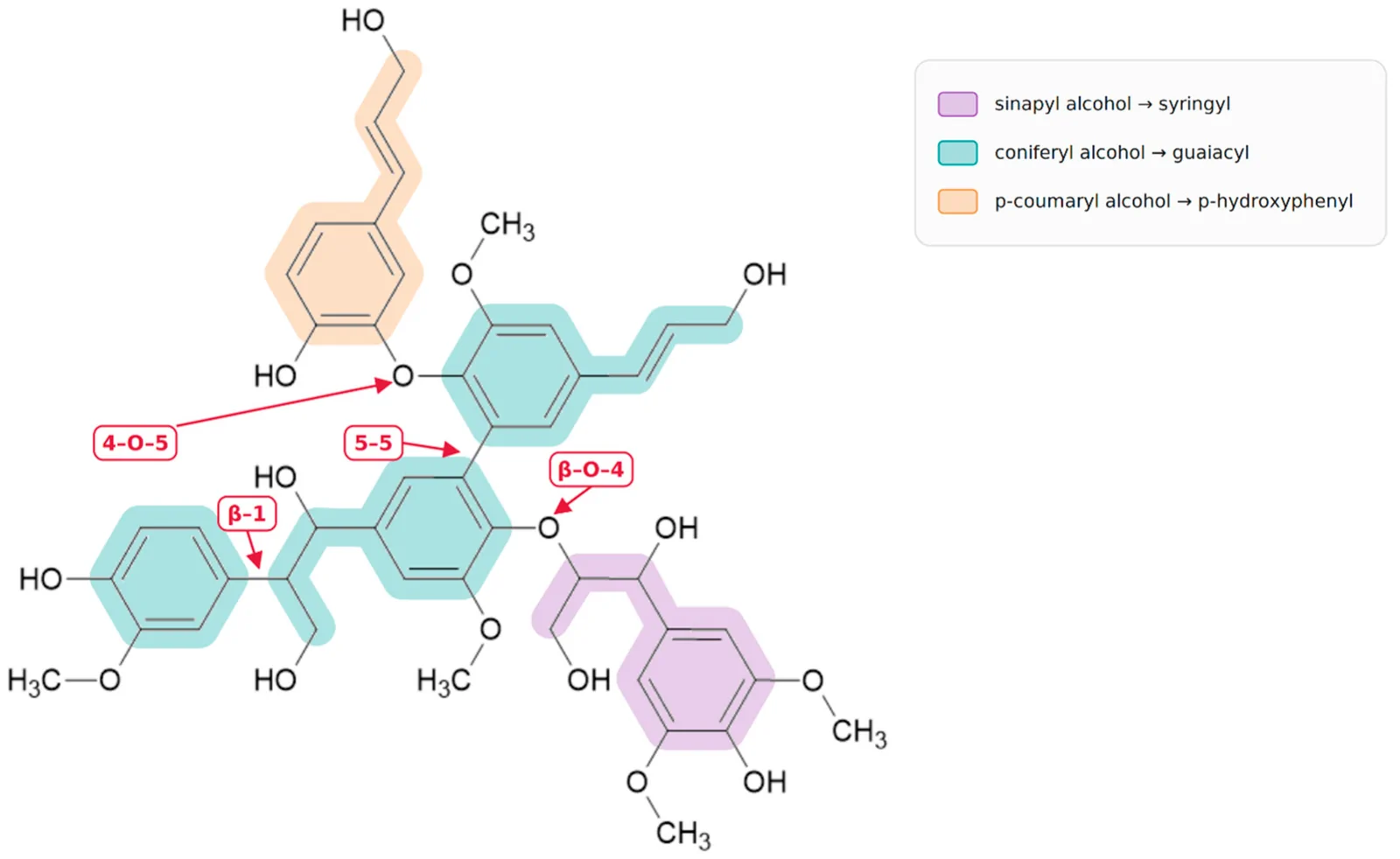
Chemical structure of a lignin oligomer, showing the three types of phenyl propanoid units and the 4-O-β, β-1, 5-5 and 4-O-5 bonds.

Lignin is a complex and sustainable natural polymer derived from waste biomass, and is the second most abundant biopolymer on Earth after cellulose [32,33]. It is composed of three phenyl propanoid units—derived from p-coumaryl alcohol, sinapyl alcohol and coniferyl alcohol-linked by 4-O-β, β-1, 5-5 and 4-O-5 bonds, according to the simplified chemical structure reported in Figure 1. The polymer, which accounts for 15% to 35% of vegetable biomass [34,35,36], is a cell-wall component acting as the binder required to give resistance and rigidity to the plant structure. In gymnosperms, lignin is synthesized by the dehydrogenative polymerization of both p-coumaryl alcohol and coniferyl alcohol, while in angiosperms sinapyl alcohol is also involved [5].

Recent advances in NMR and mass spectrometry have refined our understanding of lignin’s covalent structure. Quantitative gel-phase 2D-HSQC_0_ analysis of whole plant cell walls supports a predominantly linear, highly heterogeneous polymer in which β–O–4 aryl ethers and smaller proportions of condensed β–β, β–5, 5–5 and 4–O–5 linkages are generated through combinatorial radical coupling [37]. This work also demonstrated that lignin isolation can cleave or modify native bonds, meaning that structures measured in extracted lignins may not fully represent the polymer in planta. More recently, combined NMR and acetylation-assisted MALDI-TOF MSⁿ enabled linkage progressions to be assigned within individual spruce milled-wood-lignin oligomers, providing molecular-level information that is obscured by conventional average structural models [38]. Nevertheless, some detected structures may arise from ball milling or ionization, emphasizing the importance of distinguishing native linkages from preparation-induced artifacts [38].

Using solid-state NMR and dynamic nuclear polarization, Kirui et al. [39] showed that lignin–carbohydrate organization varies among grasses, hardwoods and softwoods. Lignin preferentially associates with xylan in non-flat conformations through non-covalent interactions, but substantial lignin–cellulose contacts also occur in woody plants. In spruce, lignin and polysaccharides exhibit homogeneous mixing at the molecular level. An atomistic model of the secondary cell wall in *Populus* wood, informed by solid-state NMR, further suggests that cellulose, hemicelluloses, and lignin assemble into a complex macromolecular architecture involving close intermolecular contacts [40]. During stem maturation, these associations become progressively denser: acetylated xylan preferentially interacts with syringyl-rich lignin, whereas methylated pectin is associated with guaiacyl-rich lignin during earlier stages of lignification [41]. Thus, lignin’s supramolecular architecture depends not only on lignin content but also on monomer composition, carbohydrate partners and developmental stage.

Current evidence indicates that lignin is a multifunctional, spatially regulated cell-wall polymer rather than merely a passive structural component. In vascular tissues, it strengthens secondary walls, limits wall hydration and prevents xylem collapse under the negative pressures generated during water transport. Its biological function depends not only on total lignin content but also on monomer composition and molecular interactions with cell-wall carbohydrates, which influence tissue mechanics [41]. Precisely localized lignin deposits also form selective apoplastic barriers: recent work in tomato demonstrated that a polar lignin cap in the root exodermis restricts passive solute movement and contributes to the regulation of mineral uptake [42]. Lignification therefore integrates mechanical support, hydraulic conductivity, solute homeostasis and protection against environmental and biological stresses.

### 2.2. Lignin Biosynthesis

Lignin biosynthesis is tightly integrated into plant primary and secondary metabolism. Its carbon skeletons originate from photosynthetically fixed carbon, which supplies glycolysis and the pentose phosphate pathway with the precursors required for the shikimate pathway and the production of phenylalanine. Lignin formation then proceeds through the phenylpropanoid pathway, beginning with the deamination of phenylalanine by phenylalanine ammonia-lyase (PAL) to yield cinnamic acid. Successive hydroxylation, methylation and CoA-activation reactions, catalysed by enzymes including C4H, 4CL, HCT, C3′H, CCoAOMT, F5H and COMT, generate hydroxycinnamoyl-CoA intermediates. These reactions depend on metabolic cofactors such as ATP, NADPH and *S*-adenosylmethionine, thereby linking lignification to cellular energy, redox and one-carbon metabolism [43].

Hydroxycinnamoyl-CoA esters are subsequently reduced by cinnamoyl-CoA reductase and cinnamyl alcohol dehydrogenase to produce the principal monolignols:p-coumaryl, coniferyl and sinapyl alcohols. These precursors give rise predominantly to the H, G and S units of lignin, respectively. Monolignols are exported from lignifying cells—or, in some tissues, supplied by neighbouring parenchyma cells—although their transport mechanisms remain incompletely resolved [44]. In the prevailing model, monolignols reach the apoplast and are oxidized by laccases and class III peroxidases. The resulting radicals undergo largely non-enzymatic combinatorial coupling, forming the heterogeneous lignin polymer within the secondary cell wall [45].

A recent study has proposed, based on lignin–carbohydrate nanoparticles isolated from differentiating spruce xylem, that these complexes may form in the Golgi or Golgi-derived vesicles and may possess a carbohydrate-rich surface surrounding an aromatic core [46]. A previous study showed that lignin forms hydrophobic nanodomains associated mainly with xylan, but this does not establish their formation in the Golgi [39]. These observations are of more than botanical interest: they would suggest that the plant itself assembles lignin in a nanoparticulate, carbohydrate-stabilised form, which is precisely the morphology that has to be reconstructed artificially when technical lignin is converted into nanolignin.

### 2.3. Industrial Production

Industrially, lignin is obtained by different processes, such as:

1. *Acidic pulping*, carried out with sulphite/bisulphite in sodium hydroxide, which yields a product composed of lignosulphonate and degraded carbohydrates.
2. *Alkaline pulping* (Kraft process), carried out with sodium hydroxide and sodium sulphide, which yields a black liquor from which chemicals and energy are recovered.
3. *Steam explosion processing* at 200 °C and high pressure, by which low-molecular-weight lignin is obtained.
4. *Organosolv pulping*, using water and solvents, which yields ethanol, cellulose and very pure lignin.

The choice among these routes is not a matter of yield alone. Each process leaves a different chemical signature on the polymer: kraft lignin carries residual sulphur and condensed structures, lignosulphonates are water-soluble and highly charged, organosolv and steam explosion lignins are sulphur-free and of lower molecular weight but vary widely with the feedstock. Molecular weight, phenolic hydroxyl content, solubility and the residual carbohydrate fraction all follow from the isolation route, and they in turn govern whether a given lignin can be dispersed into stable nanoparticles, how much charge those particles carry, and how strongly they will bind a cationic partner [20,21]. Any discussion of lignin-based carriers which does not specify the lignin source is therefore incomplete. An example relevant to the carrier systems discussed here is the Biolignin used by Coltelli et al., for which an average molecular weight of 980 g mol^−1^ was reported [28]. This value characterizes the particular lignin preparation, rather than the mass of the nanolignin particles assembled from its constituent molecules.

Lignin obtained industrially from biomass, and mainly from wood pulp and paper processing, had a market size estimated at USD 1.37 billion in 2025 and is projected to reach USD 2.04 billion by 2033, growing at a Compound Annual Growth Rate (CAGR) of 5.2% from 2026 to 2033 [6].

As mentioned previously, the different manufacturing routes do not all yield the same grade of material. Lignosulphonates and kraft lignin, which account for most of the volume produced, carry sulphonate or sulphur residues and a variable ash and carbohydrate content that restrict their use to technical applications such as binders, dispersants and fuel. The sulphur-free lignins obtained by organosolv and steam explosion, further refined by fractionation or purification, constitute instead the smaller high-purity segment on which medical, nutraceutical and cosmetic applications depend. High-purity lignin, in fact, has a continuously growing market, with a value of USD 370.8 million in 2025 and a forecast of USD 528.7 million by 2032, corresponding to a CAGR of 5.2% over the period 2025–2032 [47]. This lignin type is normally used to make binders and adhesives, phenol derivatives such as vanillin and ferulic acid, as well as specialized cosmeceuticals, dietary supplements and other bio-based products that, as alternatives to petrol-derived compounds, promote the circular and green economy [1,2].

It is fair to add that obtaining lignin in a pure and well-defined form remains difficult. On the one hand, the chemical and enzymatic systems currently available struggle with the complex bonds connecting lignin, cellulose and hemicellulose. On the other hand, microbial systems able to degrade the lignocellulose network are known to exist in the biosphere, but we do not have sufficient knowledge of their mechanism of action, so that at the moment it is not possible to design innovative engineering processes for producing pure lignin, cellulose and hemicellulose [46,48].

### 2.4. From Technical Lignin to Nanolignin

Reducing lignin to the nanoscale changes its behaviour in three respects that matter for carrier applications: it raises the specific surface area by orders of magnitude, it exposes the phenolic and carboxylic groups that carry the negative surface charge, and it converts an irregular, poorly soluble powder into a colloidally stable aqueous dispersion. The principal production routes have been reviewed in detail [21,49] and may be grouped as follows. In *solvent-shifting* or antisolvent precipitation, lignin dissolved in a water-miscible solvent such as tetrahydrofuran, acetone or ethanol is precipitated by the controlled addition of water; the method is simple and gives spherical particles of tunable size, but it requires solvent recovery. In *pH-shifting*, lignin dissolved in alkali is precipitated by acidification, a route that avoids organic solvents altogether. *Mechanical and ultrasonic* treatments fragment lignin aggregates directly in aqueous medium, while *aerosol flow* methods produce particles over a wide size range in a continuous process. More recently, organic-solvent-free procedures operating at high mass concentration have been described, which is a necessary condition for industrial scale-up [49].

In the work of Morganti and co-workers [16,17], nanolignin was obtained from lignin supplied by CIMV (Compagnie Industrielle de la Matière Végétale, Paris, France), produced by an advanced purification method in order to have a polymer as pure as possible for use in medicine, nutraceuticals and cosmeceuticals. To increase its effectiveness and skin penetration, the polymer was brought to micro-nano dimensions by dispersion in warm alkaline water under stirring, followed by ultrasound treatment, before being used as the anionic component of the chitin nanofibril-nanolignin complexes described in Section 4.3 [16,17]. This corresponds to the pH-shifting route described above, with the advantage that no organic solvent is involved at any stage.

The word nanolignin does not indicate one unique structure. It usually refers to nanoscale particles produced by the self-assembly or chemical processing of extracted lignin.

Solid lignin nanoparticles are generally spherical, amorphous particles with compact or porous interiors. In aqueous formulations, these particles constitute a solid dispersed phase, which should be distinguished from a molecular solution of lignin [50]. They consist of aggregated lignin chains held together mainly by hydrogen bonding, hydrophobic interactions, van der Waals forces and aromatic π–π interactions. Recent evidence suggests a relatively hydrophobic, higher-molecular-weight interior, while ionizable phenolic and carboxylic groups are preferentially exposed at the water-facing surface [51].

Hollow nanoparticles or nanocapsules consist of a lignin-rich shell surrounding an aqueous or oil-filled cavity. These structures are particularly useful for encapsulating drugs, pesticides or antioxidants.

In core–shell particles lignin may form the external shell around another polymer, inorganic particle or active compound. Conversely, a solid lignin core can be coated with polymers, proteins or surfactants.

Nanofibers and nanotubes are less common elongated structures produced through electrospinning, templating or interfacial assembly, often in combination with another polymer.

Nanoparticle formation normally does not create an entirely new lignin covalent structure. Instead, heterogeneous lignin macromolecules reorganize into supramolecular aggregates. Preparation conditions strongly determine their architecture: pH-shifting generally produces smaller particles with relatively compact cores, whereas solvent–antisolvent precipitation often produces larger particles with less-dense interiors. Size can range from approximately 20 nm to several hundred nanometres [51].

**Figure 2:**
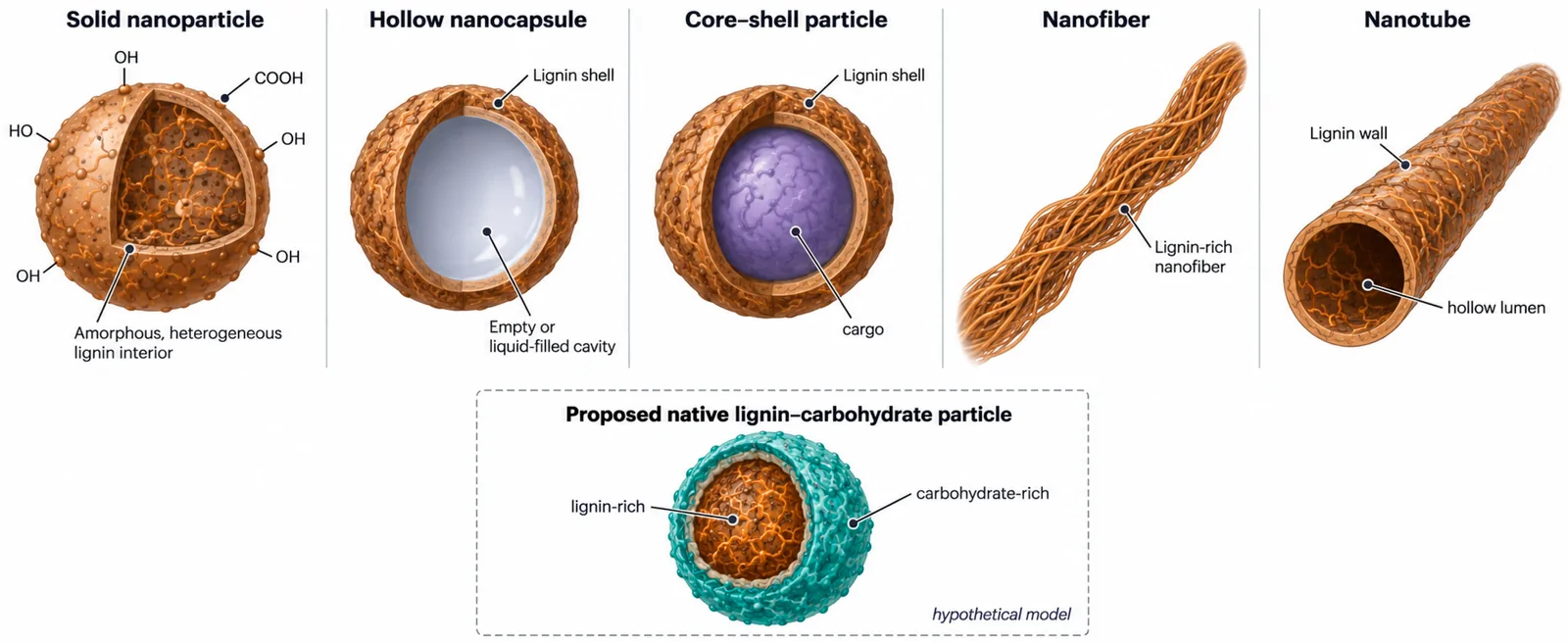
Schematic illustration of the different morphologies assumed by nanolignin as a consequence of preparation methodologies and as native lignin-carbohydrate particles in plants. Graphic generated using ChatGPT 5.5 (OpenAI).

These engineered particles should not be confused with the recently proposed native lignin–carbohydrate nanoparticles in plants, which may have a carbohydrate-rich shell and aromatic core but remain a debated biological model [46]. The different nanolignin morphologies are summarized in Figure 2.

### 2.5. UV-Screening and Antioxidant Properties

The relevance of nanolignin to carrier design is not limited to its particle architecture and colloidal behavior. Lignin has been investigated as a broad-spectrum UV-screening agent because of the chromophore and auxochrome groups present in its structure [52,53,54,55]. Vanillin and ferulic acid have shown good absorption capacity in the UVB range (280–320 nm), while lignin dimers can absorb efficiently in the UVA range (320–400 nm). This UV-blocking capacity has been exploited in specialized cosme-nutraceuticals, transparent food-packaging films, paints and varnishes [52,54]. Nanostructured lignin has also been reported to act simultaneously as an antioxidant and a UV-shielding ingredient in sunscreen formulations [53]. These properties provide a rationale for using nanolignin as a carrier component that may contribute photoprotective and antioxidant functions of its own, rather than serving solely as a scaffold for other active ingredients.

## 3. Chitin: Structure, Sources, Nanofibrils and Functional Properties

### 3.1. Structure and Biological Function

Chitin is a semicrystalline solid composed of linear polysaccharide chains built from N-acetylglucosamine (2-acetamido-2-deoxy-β-D-glucopyranose) units joined by β-1,4 glycosidic bonds [56]. It is the nitrogen-containing analog of cellulose, from which it differs only in the replacement of the hydroxyl group at C2 by an acetamido group. In the ideal polymer, every unit is acetylated, meaning that chitin is often described as a homopolymer.

**Figure 3:**
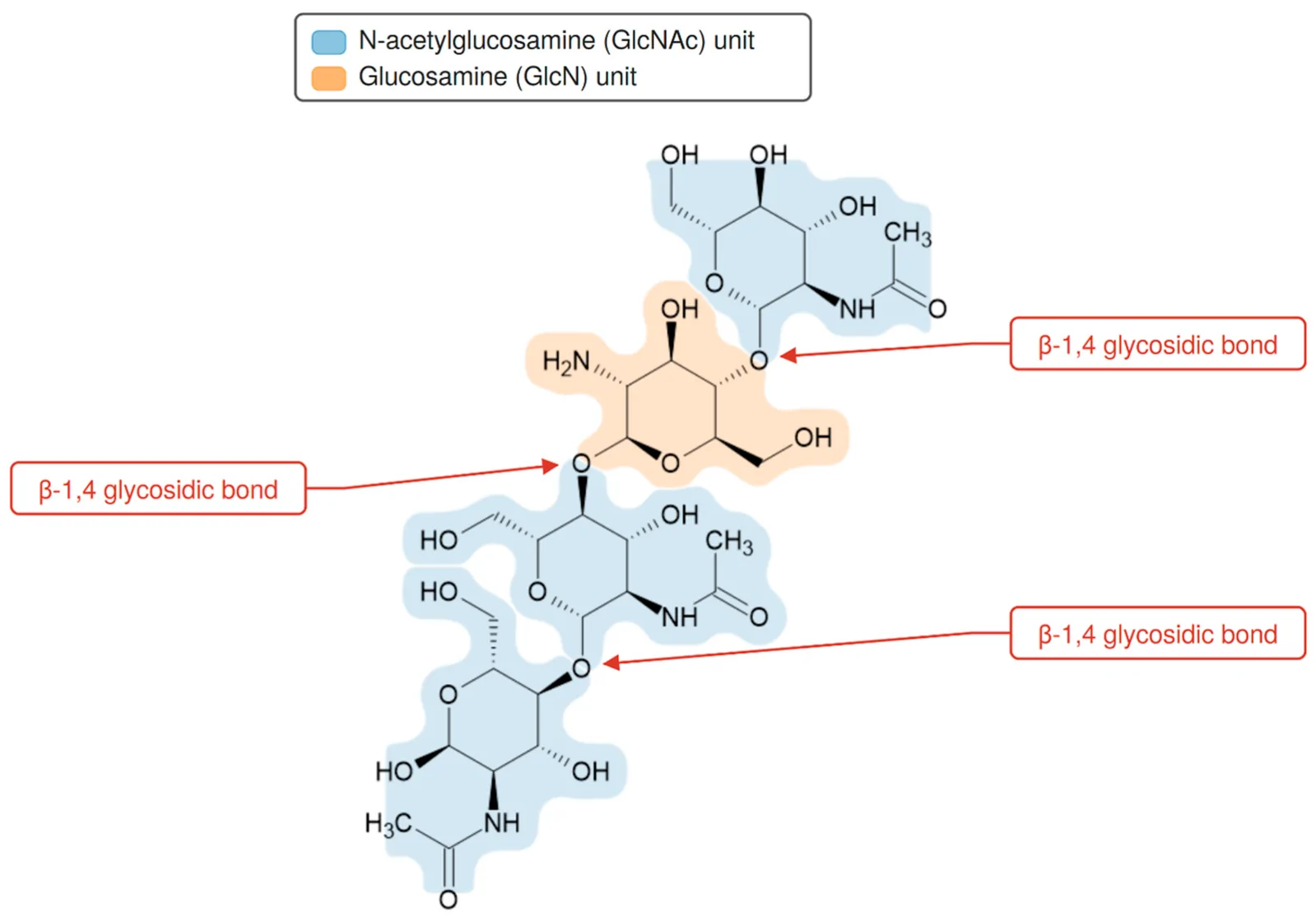
Chemical structure of a chitin oligomer showing three N-acetylglucosamine units and one glucosamine unit, as well as the β-1,4 glycosidic bonds.

However, real chitin always contains a minority of units that have lost the acetyl group: enzymatically in living organisms, through chitin deacetylases of fungi and insects, and hydrolytically during the alkaline steps of extraction [57]. The degree of acetylation of isolated chitin is therefore typically around 0.90, so that some 5–15% of the units carry a free primary amine [57,58]. Figure 3 shows a short segment of such a chain, including one deacetylated unit. When deacetylation is carried further—conventionally beyond about half of the units, at which point the polymer becomes soluble in dilute acid—the material is called chitosan.

From a strictly chemical perspective, fully acetylated chitin is a linear homopolysaccharide composed of β-(1→4)-linked N-acetyl-D-glucosamine (GlcNAc) units. Partial deacetylation introduces D-glucosamine (GlcN) residues along the polymer chain; therefore, partially deacetylated chitin and chitosan can be more accurately described as binary copolymers of GlcNAc and GlcN. Their physicochemical properties depend not only on the average degree of acetylation but also on the distribution of acetylated and deacetylated units, which may be random, block-wise or more regularly arranged [59,60]. This copolymeric interpretation is also supported by studies of chitin and chitosan obtained from the black soldier fly, *Hermetia illucens*. In particular, Coltelli et al. related differences in thermal degradation and mechanical behaviour to the relative abundance and molecular arrangement of GlcNAc and GlcN units, showing that deacetylation modifies intermolecular hydrogen bonding, chain organization and material performance [61]. Chitin and chitosan should therefore be regarded as members of a continuous structural family: highly acetylated, poorly soluble materials are conventionally classified as chitin, whereas more extensively deacetylated and acid-soluble polymers are classified as chitosan.

Although small, in chitin this deviation from the ideal homopolymer governs the charge of real chitin and has important implications. The acetamido group is not ionisable, so a perfectly acetylated chitin chain would be electrically neutral and could not take part in polyelectrolyte complexation at all. A positive charge appears only where free amino groups are present, and only when the pH is below their pKa, which lies at about 6.3–6.5 [62]. Native chitin thus carries a small and latent positive charge, expressed only in acidic media; raising that charge deliberately, by partial deacetylation of the surface, is one of the standard routes to dispersible nanochitin described in Section 3.6.

Like lignin in plants, chitin serves as the structural binder of the organisms that synthesize it. In crustaceans and insects, it forms the fibrous scaffold of the exoskeleton, mineralised with calcium carbonate and associated with proteins; in fungi, it forms the fibrils of the cell wall, covalently associated with β-glucans. In all these tissues the polymer is organised hierarchically: individual chains hydrogen-bond into crystalline nanofibrils of the order of 3 nm in diameter, which bundle into progressively coarser fibers and lamellae. This native architecture is what makes the top-down preparation of nanochitin possible, since the nanoscale elements are already present in the raw material and need only to be liberated. Three crystalline polymorphs are recognised: α-chitin, with antiparallel chains, the most abundant and most tightly hydrogen-bonded form, found in crustacean shells and fungal cell walls; β-chitin, with parallel chains, found in squid pen, which is more readily swollen and dispersed; and the rarer γ form, in which both arrangements occur [58]. Estimates of the quantity synthesized annually in the biosphere range from about 10^10^ to 10^11^ tons, placing chitin second only to cellulose among renewable polymers [63].

### 3.2. Native Chitin Morphologies

Insect cuticles generally contain tightly packed α-chitin nanofibrils embedded in a protein-rich matrix. The fibrils are arranged in successive lamellae, which may rotate gradually between layers to generate a helicoidal or “twisted-plywood” architecture [64]. This organization combines low weight with strength, flexibility and resistance to crack propagation. The degree of acetylation can also vary through the cuticle: recent measurements found more extensively deacetylated chitin in the exocuticle and highly acetylated chitin in the endocuticle.

The insect gut has a different morphology. A 2026 study showed that the peritrophic matrices of *Manduca sexta* and *Zophobas morio* contain highly acetylated, hydrated β-chitin rather than the compact α-allomorph found in the external cuticle. This more open structure is appropriate for a flexible and permeable intestinal barrier [65].

Crustacean exoskeletons also contain primarily α-chitin, but their nanofibrils are commonly organized into strongly developed Bouligand structures and embedded in proteins and calcium carbonate. Consequently, crustacean cuticle is generally more mineralized and rigid than insect cuticle. Its organization varies between the hard exocuticle and the thicker, less mineralized endocuticle.

Fungal chitin is also predominantly α-chitin, but it does not normally form a mineralized exoskeleton. Short, stiff chitin microfibrils are integrated with β-(1→3)- and β-(1→6)-glucans to form an inner load-bearing network, while mannoproteins constitute the more external fibrillar layer. In *Candida albicans*, the chitin–glucan framework behaves as a flexible molecular cage: relatively rigid chitin fibrils are connected through more deformable glucan-rich regions, allowing the wall to withstand turgor while still expanding during growth [66]. Different fungal chitin synthases can also produce microfibrils with different dimensions and localization—for example, at lateral walls, growing tips or septa.

Thus, the principal contrast is that arthropod chitin commonly forms ordered, protein-associated lamellae—often mineralized in crustaceans—whereas fungal chitin forms a non-mineralized, cross-linked microfibrillar network with glucans. The biological source determines not only α/β packing but also fibril orientation, acetylation, hydration and association with proteins, minerals or other polysaccharides. Differences in crystalline morphology and biological source are summarized in Table 1.

### 3.3. Biosynthesis of Chitin

Despite the diversity of chitin-producing organisms, its biosynthesis follows a broadly conserved mechanism. Cytosolic metabolism supplies uridine diphosphate *N*-acetyl-D-glucosamine (UDP-GlcNAc), which membrane-integrated chitin synthases polymerize into linear β-(1→4)-linked GlcNAc chains and simultaneously translocate across the plasma membrane [67].

In fungi, multiple chitin-synthase isoenzymes are differentially regulated and targeted to sites of polarized growth, septum formation and cell-wall repair, where the chains assemble into crystalline microfibrils [67]. In fungi chitin can be linked to glucan chains and for this reason it was found that the chitin nanofibrils obtained from fungi were found to be less hydrophilic than the chitin nanofibrils obtained from shrimps [68].

Arthropods use specialized enzymes for tissue-specific deposition: recent work in *Drosophila melanogaster* confirmed that chitin synthase A produces cuticular chitin in epidermal and tracheal tissues, whereas nanofibrils obtained by chitin synthase B generates the chitinous peritrophic matrix lining the midgut; these enzymes are not functionally interchangeable [69]. In molluscs, chitin synthases participate in shell formation by producing an organic scaffold that directs mineral deposition; some molluscan enzymes contain an unusual myosin motor-like domain, potentially coupling chitin synthesis to cytoskeletal transport and spatial organization [70].

Chitin biosynthesis also occurs in certain algae and protists. In the diatom *Thalassiosira pseudonana*, a specific chitin synthase localizes around the silicified girdle bands, suggesting coordinated deposition of chitin and biosilica [71]. Thus, the catalytic reaction is conserved, whereas enzyme localization, accessory domains and extracellular assembly determine the distinct biological structures produced by different organisms.

### 3.4. Sources and Production

Commercially, chitin is obtained almost entirely from crustacean shell waste—shrimp, crab and lobster—in which it accounts for between about 8% and 40% of the dry shell, the remainder being mineral matter and protein [63]. Isolation requires demineralization in dilute hydrochloric acid to dissolve the calcium carbonate, and deproteinization in dilute sodium hydroxide, optionally followed by decoloration. As with lignin, these steps are not neutral with respect to the product: they consume acid and alkali, and they inevitably cause a degree of deacetylation and chain scission, so that molecular weight, degree of acetylation and residual protein content all depend on the isolation route as much as on the biological source [57]. Any specification of a chitin grade that does not state its origin and processing is therefore incomplete. For example, α-chitin preparations obtained by controlled acid hydrolysis have been characterized with weight-average molecular weights of 0.1–1.2 × 10^6^ g mol^−1^ [72]. These values refer to the constituent polymer chains, not to the masses of nanocrystals or nanofibrils, and describe the preparations investigated rather than a universal molecular-weight range for chitin.

Two alternative sources are attracting increasing attention. Insect farming, now expanding for feed protein, yields chitin-rich byproducts with a chitin content of roughly 5–35%, available all year rather than seasonally [9]. Fungi offer a second route, which is in fact the oldest one historically: chitin was first isolated in 1811 from mushroom cell walls by Henri Braconnot and for this reason was initially named *fungine* [9,63]. Fungal cell walls contain chitin covalently associated with glucans, as a chitin–glucan complex, and mushroom cultivation generates large quantities of stalk and substrate waste, some 5 kg for every kilogram of mushrooms grown, against a world production of the order of 40 million tons a year [9]. Fungal chitin needs no demineralisation, since the cell wall is not calcified; it is more uniform in particle size and lower in molecular weight than crustacean chitin; and it avoids shellfish allergen issues, which are relevant to cosmetic and nutraceutical products [9,73]. Against these advantages, it is harder to separate from the associated glucans and is at present produced on a far smaller scale.

It should be noted that chitin from crustaceans, insects and fungi shares a β-(1→4)-linked N-acetylglucosamine backbone, but differs in its supramolecular organization and associated matrix (Table 1). In crustacean shells, predominantly α-chitin forms a composite with proteins and calcium carbonate, whereas insect cuticle contains chitin–protein fibres organized in layered, often helicoidal architectures that contribute to a range of mechanical properties. In fungi, mainly α-chitin is integrated with β-glucans and other cell-wall components. Accordingly, differences in the properties of chitin from these sources reflect both matrix composition and processing history [74,75]. In particular, the molecular weight of chitin varies across species and is strongly affected by extraction and measurement methods; consequently, source-based differences should be interpreted cautiously unless samples have been processed and characterized under comparable conditions. Nevertheless, chitosan from *Hermetia illucens* insects has generally been reported to have lower molecular weights than crustacean-derived chitosan, although the reported ranges overlap and depend on species and processing conditions [76]. In a direct comparison, Triunfo et al. found a lower viscosity-average molecular weight for *H. illucens* chitosan (obtained by chitin deacetylation) than for commercial shrimp-derived chitosan [77]. Fungal chitin is associated with β-glucans, which have been reported to reduce the hydrophilicity of chitin derivatives. Consistent with this, Panariello et al. observed greater affinity of fungal chitin nanofibrils for PBSA/PHBV films compared with shrimp-derived nanofibrils, attributing it to the fungal material’s lower polarity; this supports a source-dependent difference in surface interactions, although it is not a direct measurement of hydrophilicity [68].

### 3.5. Chitin Nanocrystals

Chitin nanocrystals are rod-like particles obtained by removing the amorphous domains of the polymer and leaving the crystalline ones intact. The classical route is acid hydrolysis, typically in 3 M hydrochloric acid at 85–105 °C for one to three hours, followed by washing and centrifugation to remove the acid, dialysis, and ultrasonication to break up the aggregates formed on drying [78]. The amorphous regions, being more accessible to the acid, are hydrolysed preferentially, so that what survives is a suspension of highly crystalline rods. For α-chitin from shrimp or crab, the particles so obtained are commonly 10–15 nm in diameter and 200–500 nm in length [78].

Because the hydrolysis attacks the polymer rather than merely disassembling it, this route is costly in acid and in yield, and much recent work has been directed at improving both; pretreatment with hydrochloric acid vapour, for example, has been reported to raise yields substantially by exfoliating the amorphous material without over-hydrolysing the crystalline fraction [79].

The residual amino groups exposed at the crystal surface are protonated in the acidic medium in which the hydrolysis is carried out, so the crystals emerge with a positive surface charge and a hydration shell that keeps them dispersed. In the colloidal suspension obtained by Morganti and co-workers, each crystal had a mean size of 240 × 7 × 5 nm and a mean weight of 0.074 × 10^−6^ ng, at a pH between 2 and 4 [7]. It is this positive charge—latent in the native polymer and expressed in the acidic suspension—that distinguishes nanochitin from almost every other natural nanopolymer, cellulose nanocrystals and nanolignin included, and that allows it to form the complexes that will be described in Section 4.2 and Section 4.3.

### 3.6. Chitin Nanofibrils

A nanofibril is conventionally defined as a fibre less than 100 nm in diameter with an aspect ratio above 100 [80,81]. Chitin nanofibrils are obtained by disassembling the native hierarchical chitin structure mechanically, rather than by dissolving away its amorphous parts chemically. The consequence is a longer, more entangled, less crystalline particle that retains the amorphous domains and forms network gels at low solid content, in contrast to the short, stiff, highly crystalline rods obtained by acid hydrolysis.

The disintegration is performed with grinders, high-pressure homogenisers or waterjet systems, by ultrasonication, or by aqueous counter-collision [80,82]. Whichever device is used, the mechanical energy has to overcome the dense hydrogen-bond network that holds the fibrils together, and it succeeds only if the fibrils also repel one another electrostatically. For unmodified native chitin this means working in acidic conditions, where protonation of the surface amino groups charges the fibrils positively; without that repulsion the material merely fragments into coarse aggregates [80]. Surface charge is therefore not an incidental property of nanochitin but the enabling condition for its preparation.

The positive surface charge density of native chitin can be increased by partial deacetylation with sodium hydroxide at elevated temperature. This increases the number of primary amine groups along the polymer chain and, after mechanical treatment, yields cationic fibrils of about 6 nm in diameter with high aspect ratio [82]. An alternative approach, based on TEMPO-mediated oxidation, converts the hydroxymethyl groups (–CH_2_OH) carried by C6, i.e., the exocyclic carbon of each sugar unit, into carboxylate units (–COO^−^). The resulting nanofibrils, unlike those obtained by partial deacetylation, are negatively charged and have a diameter of about 3–15 nm [83]. It should be clear that the two approaches are not equivalent for the purposes of this review: TEMPO-oxidised nanochitin is anionic and cannot play the same role as cationic nanochitin in the formation of the complexes that will be discussed in Section 4.3.

### 3.7. Potential Role of Chitin Nanofibrils in Cutaneous Hyaluronan Synthesis

**Figure 4:**
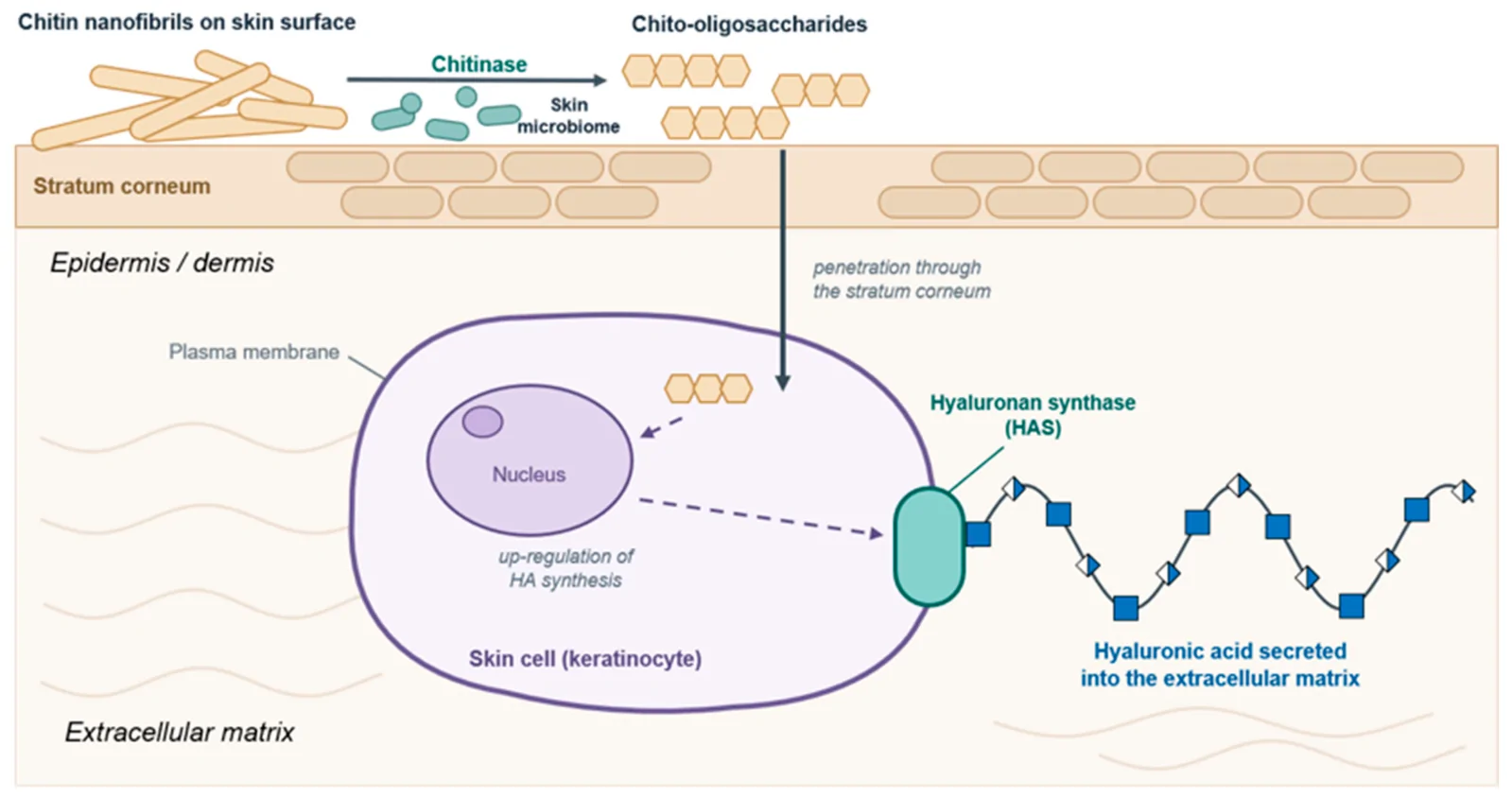
Hypothetical route by which topically applied chitin nanofibrils may support cutaneous hyaluronan synthesis. At the skin surface, chitinase produced by the resident microbiota hydrolyzes the nanofibrils into short chito-oligosaccharides. These traverse the stratum corneum and are taken up by cells of the viable epidermis, where they up-regulate hyaluronan synthase. The enzyme, embedded in the plasma membrane, extrudes the growing hyaluronan chain directly into the extracellular matrix.

In addition to the surface-charge properties discussed above, chitin nanofibrils are of interest for their potential biological activity in dermocosmetic applications. It has been hypothesized that chitinases produced by the skin microbiota can degrade the nanofibrils into short chito-oligosaccharide fragments. Following uptake by cutaneous cells, these fragments may stimulate hyaluronan synthase activity, thereby promoting the production and release of hyaluronic acid into the extracellular matrix (ECM), as illustrated in Figure 4 [84,85,86,87]. Hyaluronic acid is a highly hydrophilic ECM polysaccharide whose capacity to bind and retain water contributes to tissue hydration and viscoelasticity [88]. Through interactions with cell-surface receptors such as CD44, it also participates in the regulation of keratinocyte proliferation and differentiation, contributing to epidermal renewal and maintenance of the skin’s protective barrier [89,90].

Chitin complexes have also been associated with anti-aging activity exerted from the inside out [91] and with the development of pro-aging cosme-nutraceuticals [92]. The proposed chitin–hyaluronan pathway provides a rationale for investigating chitin nanofibrils as potentially active carrier components, rather than solely as structural scaffolds.

## 4. Polyelectrolyte Complexation of Nanopolymers

Combining the functional properties of nanolignin and chitin nanofibrils in a single carrier requires an understanding of the interactions that govern their assembly. When a polymer is structured at nanometric dimensions, its behavior becomes increasingly influenced by its interfacial properties: the high surface-area-to-volume ratio makes the chemical groups and charges exposed at the interface particularly important in controlling colloidal stability, interactions and reactivity. Two nanopolymers carrying opposite charges, therefore, do not simply mix: they self-assemble. When they meet in an aqueous medium, the small counterions condensed along each chain are released into the bulk solution, and the entropy gained in this way, together with the electrostatic attraction between the two macroions, drives the spontaneous formation of the so-called polyelectrolyte complexes [93,94]. Secondary interactions—hydrogen bonds between hydroxyl groups, hydrophobic and aromatic interactions between the apolar domains—contribute to the cohesion of the resulting structure.

Depending on the charge ratio, pH, ionic strength, the molecular weights of the two polyelectrolytes, and the order of mixing, the assembly may take the form of soluble complexes, coacervates, compact colloidal particles, precipitates or capsule-like structures [95,96]. Two consequences of this dependence deserve some comments. First, at a charge ratio close to unity, the complex is electrically neutral and tends to aggregate, so that a deliberate excess of one of the polyelectrolytes is normally used to maintain dispersion stability; the sign of the residual charge then determines whether the net surface charge of the resulting particles is positive or negative [97]. Second, because the interaction is non-covalent, it is reversible: an increase in ionic strength screens the electrostatic attraction and may weaken or dissociate the complex, which is a potential disadvantage for long-term stability but may be exploited to achieve triggered release.

From the formulator’s point of view, the interest of polyelectrolyte complexation of nanopolymers lies above all in what it does not require: no organic solvents, no chemical cross-linkers, no surfactants and no high-energy input, the whole process taking place in water at room temperature. Active ingredients can therefore be incorporated during self-assembly, allowing the preparation of carrier systems under comparatively mild aqueous conditions [27,28]. Appropriate complex design can improve the stability of active ingredients and provide gradual or stimulus-responsive release at the intended site and/or under the desired conditions [27,28,97].

### 4.1. Carriers Based on Lignin Nanoparticles

Lignin nanoparticles have been investigated as carriers for pharmaceuticals, pesticides, enzymes and biological macromolecules, and the field has been critically reviewed [20,21]. Being anionic, colloidal lignin particles spontaneously adsorb polycations and proteins: gelatin and poly-L-lysine were found to form the most pronounced corona, and the relative weight of the electrostatic, hydrophobic, van der Waals and hydrogen-bonding contributions proved to depend on the ionic strength of the medium [22]. Lignin nanoparticles coated in this way with poly-L-lysine, poly-L-glutamic acid or beta-casein have been used as adhesives for soft materials and to modify cellulose nanofibrils [98], while the adsorption of a cationic lignin onto anionic lignin particles reversed their surface charge and produced durable Pickering emulsions in an all-lignin system [99].

Despite substantial progress in lignin-based nanocarriers, several unresolved issues limit comparisons across studies and translation into practical applications. A central challenge is the intrinsic heterogeneity of lignin. Differences in botanical origin, extraction conditions, molecular-weight distribution, and functional-group composition can influence nanoparticle formation and interactions with encapsulated compounds [29,50,100]. Consequently, detailed characterization of the starting lignin is essential for establishing reproducible relationships between carrier structure and performance.

Carrier performance also requires assessment beyond encapsulation efficiency alone. High encapsulation efficiency does not necessarily imply high loading capacity or adequate release, since cargo retention and release depend on both lignin–cargo interactions and carrier architecture [29,30]. These parameters should therefore be quantified separately and evaluated in relation to the intended application. Similarly, release profiles must be interpreted in the context of the experimental medium. Studies of budesonide-loaded particles, cross-linked lignosulfonate carriers, and esterified lignin nanoparticles illustrate how pH, surfactants, cross-link density, and the surrounding phase can influence release behavior [29,30,31]. Direct comparisons between formulations tested under different conditions should therefore be approached cautiously.

Another important consideration is the contribution of the carrier itself to the observed response. Recent work on everolimus-loaded lignin nanoparticles suggests that lignin-derived components may contribute to biological activity alongside the delivered drug, although the proposed mechanisms require further validation [101]. Appropriate controls—including unloaded nanoparticles, the free active compound, and their physical mixture—are therefore needed to distinguish the effects of encapsulation from those of lignin. Finally, practical implementation depends on whether laboratory-scale advantages can be maintained during production, storage, and use. Solvent recovery, purification requirements, colloidal stability, degradation behavior, and application-specific safety must be considered alongside delivery performance [20,49]. Addressing these interconnected challenges will help establish clearer links between lignin chemistry, particle assembly, cargo retention, release mechanisms, and functional outcomes.

### 4.2. Carriers Based on Chitin Nanofibrils

On the chitin side, the literature has developed largely around emulsion stabilisation and film reinforcement rather than around encapsulation. Positively charged chitin nanocrystals and negatively charged cellulose nanocrystals mixed in water gave nanoconjugates that are electrically neutralized at a 1:1 mass ratio, with spectroscopic evidence of a direct electrostatic association between the two nanoparticles, and these conjugates stabilized oil-in-water emulsions better than either component alone and with a markedly reduced salt requirement [23].

Within the cosmetic field, chitin nanofibrils (CN) have been used in combination with hyaluronic acid (HA), giving the CN-HA complexes, and embedded in non-woven tissues for skin applications [16,17,18]. The rationale is the same in both of the above examples: the cationic nanocrystal acts as the scaffold on which an anionic polymer of cosmetic interest is deposited, the complex being formed in water without additional chemistry.

In the systems reviewed in this section and the preceding one, however, only one polyelectrolyte is a functional nanopolymer recovered from waste biomass, the other being a synthetic polyelectrolyte, a protein, a soluble polysaccharide or a structural nanocellulose. The complexes considered in the next section differ in that both polyelectrolytes are nanoparticulate, both are recovered from waste biomass and both retain a biological activity of their own.

Considering CN in carrier applications, a distinction should be maintained between chitin nanofibrils, surface-deacetylated chitin nanofibrils, chitin nanocrystals, and chitosan nanoparticles. In particular, the degree and distribution of deacetylation influence the crystalline structure and biological interactions of chitin nanofibrils, making these parameters essential descriptors of the starting material [102].

A major driver for developing chitin-nanofibril-based carriers is the possibility of combining structural reinforcement with active-compound incorporation and controlled release. Partial surface deacetylation introduces amino groups that can become protonated under acidic conditions, enabling association with negatively charged components. This approach has been exploited to construct composite systems with hyaluronic acid, anionic cyclodextrins, and oxidized cellulose nanofibers [24,25,26].

Representative studies illustrate the versatility of this strategy. Anraku et al. developed surface-deacetylated chitin nanofibril–hyaluronic acid tablets for extended famotidine release, identifying the formation of a hydrated surface barrier as a mechanism that slowed drug transport [24]. Tabuchi et al. incorporated prednisolone into chitin nanofibril–sulfobutyl ether β-cyclodextrin gels and demonstrated sustained release, with corresponding effects on drug absorption following oral administration in rats [25]. Xu et al. investigated hydrogels formed from partially deacetylated α-chitin nanofibers and TEMPO-oxidized cellulose nanofibers for 5-fluorouracil delivery, demonstrating relationships between network organization and loading–release behavior [26]. More recently, Lin et al. developed injectable, self-healing chitin nanofiber–alginate dialdehyde hydrogels for doxorubicin delivery, reporting sustained release and suppression of tumor growth in animal models [103]. These studies support the potential of nanofibril-containing matrices for controlled delivery, while their findings remain specific to the compositions and experimental models investigated.

### 4.3. Chitin Nanofibril-Nanolignin (CN-NL) Complexes

**Figure 5:**
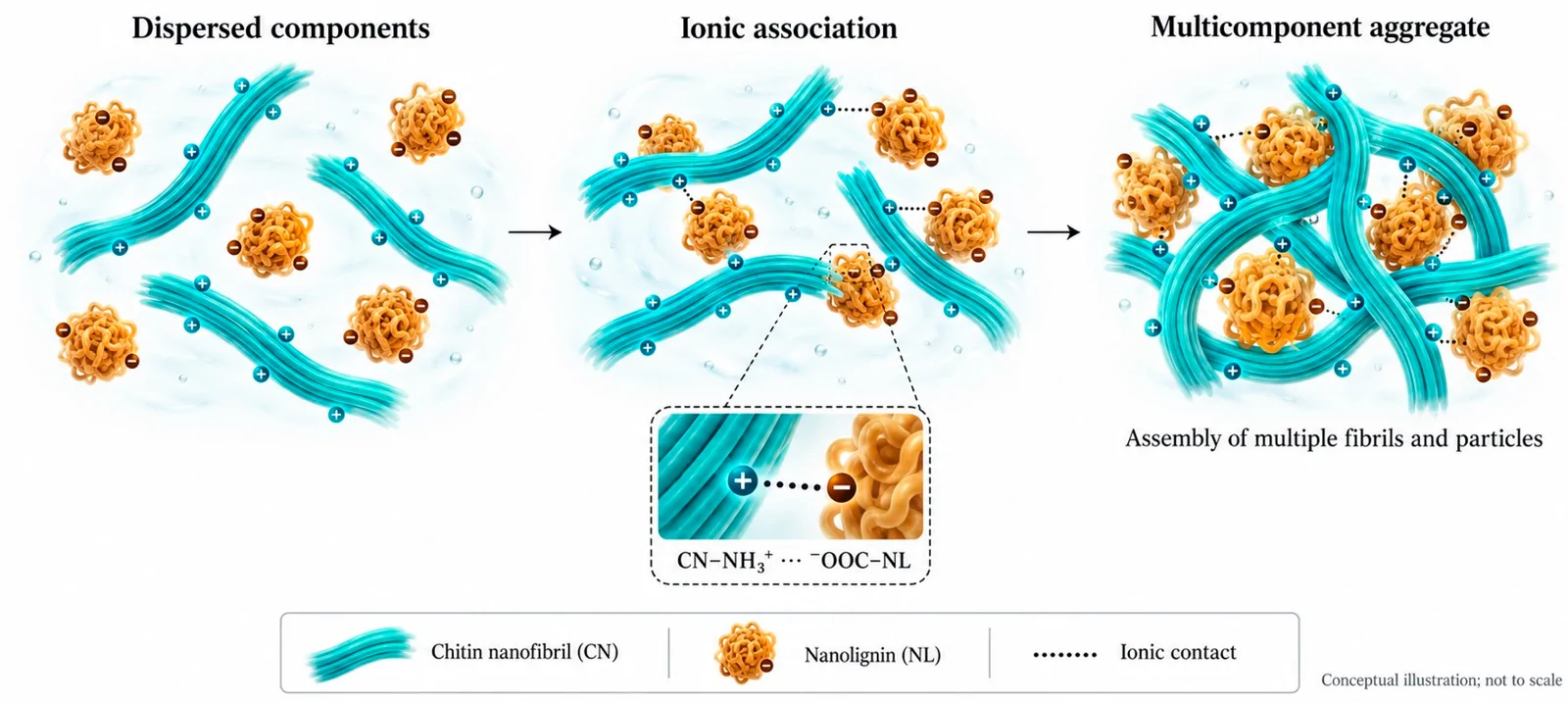
Conceptual illustration suggesting how the assembly of CN and NL through ionic interactions may occur. Scheme generated using ChatGPT (OpenAI).

Complex formation between CN and NL can be interpreted as an ionic association between protonated amino groups (–NH_3_^+^) on the surface of chitin nanofibrils and carboxylate groups (–COO^−^) on nanolignin particles. Differences in the density and accessibility of these oppositely charged sites may favor association among multiple fibrils and particles rather than discrete one-to-one complexes. Such multicomponent assembly is consistent with the observed formation of micrometer-sized aggregates (Figure 5) [28].

The system closest to the one discussed here is that of Zou and co-workers, who adsorbed a thin film of chitosan onto colloidal lignin particles [104]. Full surface coverage was obtained with only 5 wt% of chitosan relative to the dry weight of the particles, and this was sufficient to reverse the surface charge from negative to positive. The coated particles stabilized olive-oil Pickering emulsions, and the resulting microcapsules retained their shape on drying and rewetting when the chitosan corona was ionically cross-linked; encapsulation and rapid release of a model lipophilic drug were demonstrated. The authors explicitly framed the work as the replacement of synthetic polyelectrolytes by abundant biopolymers. The difference from the complexes discussed in the rest of this section is that chitosan is a soluble, deacetylated polycation, whereas chitin nanofibrils are crystalline nanoparticles that retain the acetylated structure and the biological recognition associated with it.

The preparation of the CN-NL complexes and of the tissues derived from them has been described in detail elsewhere, together with the corresponding characterization data [16,17,18,27,28,105]; only the essential features are summarised here. Chitin nanofibrils (CN) were prepared as a pure aqueous suspension in a closed-loop reflux flask, leaving the food-grade polymer for one hour in acidic water and then washing it three times with deionized water, followed by filtration and ultrasound treatment. Nanolignin (NL) was obtained by dispersing the pure polymer in warm alkaline water under stirring, as described in Section 2.4.

**Figure 6:**
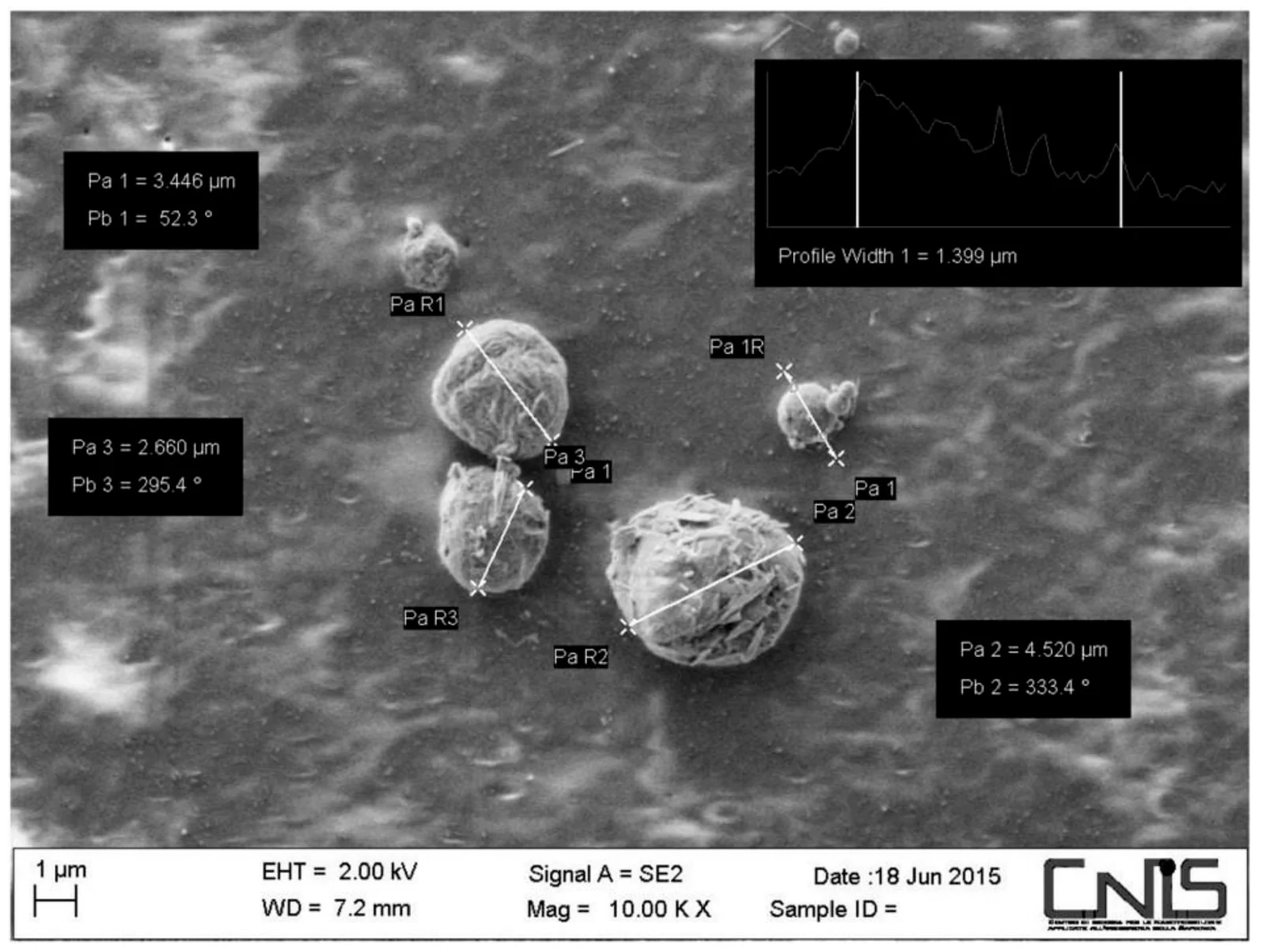
SEM micrograph of chitin nanofibril-nanolignin (CN-NL) complex particles, showing micrometer-scale assemblies containing nanoscale constituents. Reproduced from Figure 1a of [Morganti, P., Coltelli, M.-B., Gagliardini, A., Lazzeri, A., Morganti, G., Simonetti, G., … Donnarumma, G., 2023. Biopolymer- and Natural Fiber-Based Biomimetic Tissues to Realize Smart Cosmeceuticals and Nutraceuticals Using an Innovative Approach. Pharmaceutics 15 (11), 2525. doi:10.3390/pharmaceutics15112525.] [106]. © 2023 by the Authors. Licensed under CC BY 4.0. Only panel (a) is reproduced.

The alkaline nanolignin solution, pre-treated again by ultrasound, was then poured into the acidic CN suspension together with the selected active ingredients, in order to obtain the activated CN-NL microcapsules. The cited studies report a CN:NL mass ratio of 2:1, while they do not specify a pH value for the complexation step [27,28]. The morphology of CN-NL complex microcapsules is illustrated in Figure 6 [106].

In terms of the framework set out in Section 4, this is a polyelectrolyte complexation between cationic chitin nanofibrils and anionic nanolignin particles, carried out in water at room temperature, in which the active ingredient is entrapped during the assembly itself rather than loaded afterwards. The microcapsules were then poured into the gel of the selected natural polymers and electrospun to obtain an activated tissue [16,17,18,19].

This tissue-carrier, embedded with chitin nanofibril-nanolignin complexes, is able to encapsulate different active ingredients and has been shown to load, transport and release the desired agents at the intended layer of the human skin and mucous membranes. Specifically, in a human in vivo tape-stripping study, an electrospun pullulan tissue containing CN–NL complexes loaded with nicotinamide, allantoin and fish-collagen peptides was reported to yield greater stratum-corneum recovery of nicotinamide than a water-in-oil emulsion containing the same amount of nicotinamide. However, it should be noted that, due to the absence of allantoin and fish-collagen peptides in the tested emulsion, this was not a single-variable comparison [107]. Moreover, unlike conventional emulsions, this vehicle can be formulated without preservatives, emulsifiers, fragrances, colorants, or other ingredients that may cause allergic reactions or sensitization [14,15]. It may therefore be used to develop medically oriented cosme-nutraceutical products [108,109].

In addition to delivering incorporated actives, CN–NL complexes can bring together the UV-screening and antioxidant properties of nanolignin discussed in Section 2.5, and the potential contribution of chitin nanofibrils to cutaneous hyaluronan synthesis outlined in Section 3.7. The rationale is therefore that of an active carrier, whose components may contribute functions of their own rather than merely provide an inert vehicle.

An additional advantage of the solid formats is that the resulting cosme-nutraceuticals may be packed in biodegradable paper and/or aluminium foil, avoiding the plastic containers normally used, and that the water otherwise needed to make an emulsion is not required at all [11,12].

Lignin has also been incorporated into composite films to provide UV shielding for packaging applications [54].

From a formulation perspective, the systems reviewed here illustrate several product formats with different functional requirements. For topical use, nanostructured lignin has been investigated in sunscreen formulations for its UV-screening and antioxidant properties [53], while surface-modified lignin particles and chitin–cellulose nanocrystal conjugates have been used to stabilize emulsions [23,99]. CN–HA and CN–NL complexes can also be incorporated into non-woven tissues for skin-contact applications [16,17,18]. A specific example is an electrospun pullulan tissue containing CN–NL complexes loaded with nicotinamide, allantoin and fish-collagen peptides [107]. Applications involving oral administration are illustrated by nanochitin complexes carrying melatonin, vitamin E and β-glucan, formulated as topical emulsions and oral hard capsules and evaluated separately and in combination [91]. In a randomized, 12-week human study, the complexed formulations were reported to produce greater improvements in skin hydration, elasticity and clinical appearance than the corresponding formulations containing non-complexed actives [91]. Related pharmaceutical studies have also investigated nanochitin-containing tablets and gels for extended famotidine release and sustained prednisolone delivery, respectively [24,25]. Thus, the benefits of nanolignin and nanochitin differ with the application, encompassing UV screening, antioxidant activity, emulsion stabilization, matrix formation and active-ingredient delivery, and should be evaluated for the specific formulation and administration route.

Table 2 summarizes the principal applications, reported advantages and limitations of the nanopolymer complexes and related lignin- and chitin-based formulations discussed in this section.

## 5. Safety and Regulatory Status

The materials discussed in this review are natural, biodegradable and derived from waste, but these attributes do not by themselves establish either safety or regulatory acceptability, and it would be misleading to present them as though they did.

In the European Union, cosmetic products are governed by Regulation (EC) No 1223/2009, which defines a nanomaterial as an insoluble or biopersistent and intentionally manufactured material with one or more external dimensions, or an internal structure, on the scale from 1 to 100 nm [110]. Products containing nanomaterials must be notified through the Cosmetic Products Notification Portal, and those whose nanomaterials are not colorants, preservatives or UV-filters require an additional notification six months before being placed on the market. The Scientific Committee on Consumer Safety has issued guidance setting out the physicochemical characterisation, exposure assessment and toxicological data expected in a nanomaterial safety dossier [111]. Whether a given chitin nanofibril or nanolignin preparation falls within the regulatory definition depends on its solubility and biopersistence and must be established case by case; the question cannot be settled by the natural origin of the polymer. The absence of a harmonised position on biodegradable organic nanoparticles of this kind is itself a barrier to industrial adoption, and it has been identified as such in the lignin carrier literature [20]. It should be noted that this regulatory overview is provided here for informational purposes only and does not constitute legal advice.

In addition, as with many emerging technologies, there is a growing body of patent literature on lignin and nanolignin, chitin and nanochitin, and their combinations with one another or with other polymers, as well as on their preparation and industrial production methods. This may pose potential patent-related constraints on the commercial use of these materials for some applications. However, a discussion of this topic lies beyond the scope of the present scientific review.

## 6. Product Quality

### 6.1. Color

Technical lignin is brown, and the intensity of its color increases with the condensation of the structure. For a leave-on cosmetic this is a practical obstacle at least as serious as any toxicological question, and it constrains the maximum usable concentration. Fractionation and bleaching procedures yielding light-colored lignin with retained UV absorption have been developed [55] but they add cost and may reduce the phenolic content responsible for the antioxidant activity. The trade-off between color, antioxidant capacity and UV absorption has not, to our knowledge, been systematically mapped for cosmetic use.

### 6.2. Batch-to-Batch Variability

Technical lignin composition varies with feedstock, season, geographical origin and process conditions [20,21]. Since molecular weight and phenolic content govern surface charge, a change of batch may alter the stoichiometry of the complex and therefore its size, stability and release profile. Reproducible manufacture requires specification of the lignin by measured physicochemical parameters rather than by supplier and grade alone.

### 6.3. Colloidal Stability and Degradation

Polyelectrolyte complexes are held together by reversible interactions and are sensitive to ionic strength and pH; lignin particles themselves may aggregate or dissolve depending on the medium, and their stability and degradation behavior have been reviewed critically [49]. Data on the shelf life of CN-NL complexes in finished formulations, and on their fate after application, remain limited.

### 6.4. Evidence Base

Finally, much of the published work on chitin- and lignin-based cosmetic carriers, including part of that discussed here, rests on a relatively small number of studies from a limited number of groups. Independent replication, controlled clinical evaluation and standardized release testing against conventional emulsions would substantially strengthen the case for these materials.

## 7. Conclusions and Outlook

Lignin and chitin offer complementary starting points for functional biopolymer carriers. Lignin provides a heterogeneous aromatic framework whose composition and chromophores influence particle assembly, antioxidant behavior and UV attenuation. Chitin provides a semicrystalline polysaccharide scaffold that can be recovered as nanocrystals or nanofibrils, with dimensions, degree of acetylation and accessible surface groups controlling its interfacial behavior. Both polymers can therefore contribute to the design of particles, coatings, emulsion stabilizers and fibrous carriers.

This review has illustrated that biological source, isolation and nanostructuring cannot be separated from carrier performance. Technical lignins are not chemically interchangeable, and nanochitin is neither a single morphology nor an invariably cationic material. The connection between composition, processing, morphology, surface chemistry and function must be established for the actual material used.

In this context, polyelectrolyte complexation provides a useful assembly strategy. It can combine the complementary properties of lignin and chitin, or associate either polymer with other functional components under relatively mild conditions. In particular, chitin nanofibril–nanolignin complexes can be assembled in water at room temperature, without organic solvents, crosslinkers or surfactants during the complexation step. Both polymers can be recovered from waste biomass and may contribute functions beyond active-ingredient delivery: lignin can provide photoprotective and antioxidant activity, whereas chitin has been proposed to support cutaneous hyaluronan synthesis. Dry tissue carriers incorporating these complexes offer the possibility of reducing formulation water and plastic packaging relative to conventional emulsions. They can also be designed without emulsifiers or fragrances and, provided that microbiological safety is demonstrated throughout the intended shelf life and use, without preservatives.

Three lines of work appear necessary before the potential of these materials can be realized. First, the relationships among polymer source, processing, surface chemistry and carrier structure should be mapped systematically, including the roles of surface charge and composition in polyelectrolyte complexes, so that reproducible material specifications can be established. Second, the release behavior of the different carrier formats, including tissue carriers, should be quantified using standardized methods, and their stability over the shelf life of a finished product confirmed. Third, the safety and regulatory acceptability of each preparation should be assessed for its intended use. For cosmetic applications, this assessment should be supported by the characterization and toxicological data specified in the relevant guidance [110,111]. Addressing these challenges would facilitate the wider use of lignin- and chitin-based carriers in cosmetic and nutraceutical products, with potential benefits in terms of environmental sustainability and, for topical applications, skin compatibility.

## Figures and Tables

**Table 1:** Principal chitin allomorphs: chain arrangements, characteristic structural properties and representative biological sources.

Allomorph	Chain Arrangement	Main Characteristics	Representative Sources
**α-Chitin**	Adjacent chains are predominantly antiparallel	Dense hydrogen-bonding network; high crystallinity, stiffness and chemical resistance	Arthropod cuticles and exoskeletons; most fungal cell walls
**β-Chitin**	Chains are parallel	More open and hydrated structure; greater swelling, flexibility and chemical accessibility	Squid pens, tubeworms, some diatoms and insect peritrophic matrices
**γ-Chitin**	Traditionally described as a mixture of parallel and antiparallel chains	Intermediate or heterogeneous packing; less well characterized and sometimes regarded as a variant of α-chitin	Some insect structures, including particular cocoons and gut-associated matrices

**Table 2:** Complexes of nanopolymers and related lignin- and chitin-based formulations: applications, reported advantages and limitations.

Nanopolymer Complex or Related Formulation	Applications	Reported Advantages	Comments	References
Lignin nanoparticles, including modified and cross-linked carriers	Encapsulation and delivery of pharmaceuticals, pesticides, enzymes and biological macromolecules	Accommodation of diverse cargos; release behavior can be modulated through lignin–cargo interactions and carrier architecture	Lignin heterogeneity complicates reproducibility. Encapsulation efficiency, loading capacity and release must be assessed separately.	[20,21,29,30,31,50,100,101]
Lignin nanoparticles coated with proteins, polypeptides or cationic lignin	Adhesion to soft materials, modification of cellulose nanofibrils and Pickering emulsion stabilization	Adsorption modifies particle surfaces; cationic lignin coatings reverse surface charge and enable stable all-lignin Pickering emulsions	-	[22,98,99]
Chitin nanocrystal–cellulose nanocrystal conjugates	Oil-in-water emulsion stabilization	Improved stabilization compared with either component alone, with a reduced salt requirement	Comparisons between formulations tested under different conditions require caution.	[23]
CN–HA complexes incorporated into non-woven tissues	Dermocosmetic and skin-contact applications	Aqueous assembly without additional chemical reactions; incorporation of the complexes into fibrous delivery formats	-	[16,17,18]
Surface-deacetylated CN–HA tablets and CN–sulfobutyl ether β-cyclodextrin gels	Famotidine and prednisolone delivery, respectively	Extended famotidine release associated with a hydrated surface barrier; sustained prednisolone release with effects on absorption after oral administration in rats	Findings are specific to the formulations and experimental models investigated; the reported prednisolone absorption evidence is from rats.	[24,25]
Chitin nanofiber–oxidized cellulose and chitin nanofiber–alginate dialdehyde hydrogels	5-Fluorouracil and doxorubicin delivery, respectively	Injectable, self-healing doxorubicin hydrogels showing sustained release and tumor-growth suppression in animal models	Performance depends on matrix composition and organization; the reported anti-tumor findings are limited to animal models.	[26,103]
Chitosan-coated colloidal lignin particles	Olive-oil Pickering emulsions and encapsulation of a model lipophilic drug	Retention of capsule shape after drying and rewetting; rapid release of the model drug	Drying–rewetting performance was demonstrated with an ionically cross-linked corona.	[104]
CN–NL complexes incorporated into electrospun tissues	Topical delivery; a tested pullulan tissue contained nicotinamide, allantoin and fish-collagen peptides	Active ingredients incorporated during aqueous assembly; greater stratum-corneum recovery of nicotinamide than from a water-in-oil emulsion in a human tape-stripping study	The emulsion lacked allantoin and fish-collagen peptides, so the comparison does not isolate carrier effects.	[16,17,18,19,27,28,107]
Lignin-containing composite films	UV-protective packaging	Exploitation of lignin’s UV-shielding properties	-	[54]
Nanostructured lignin incorporated into sunscreen formulations	Topical sunscreen formulations	UV-screening and antioxidant activity	Benefits depend on the specific formulation.	[53]
Nanochitin complexes with melatonin, vitamin E and β-glucan	Topical emulsions and oral hard capsules for skin-care applications, used separately or together	Greater reported improvements in skin hydration, elasticity and clinical appearance than with the corresponding non-complexed actives	12-week randomized human study; findings specific to the tested multi-ingredient formulations.	[91]

## Data Availability

No new data were created or analyzed in this study. Data sharing is not applicable to this article.

## Abbreviations

The following abbreviations are used in this manuscript:

4CL	4-Coumarate:CoA ligase
ATP	Adenosine triphosphate
C3′H	p-Coumaroyl shikimate/quinate 3′-hydroxylase
C4H	Cinnamate 4-hydroxylase
CCoAOMT	Caffeoyl-CoA O-methyltransferase
CIMV	Compagnie Industrielle de la Matière Végétale
CN	Chitin nanofibrils
CN–HA	Chitin nanofibril–hyaluronic acid
CN–NL	Chitin nanofibril–nanolignin
CoA	Coenzyme A
COMT	Caffeic acid O-methyltransferase
ECM	Extracellular matrix
F5H	Ferulate 5-hydroxylase
GlcN	D-Glucosamine
GlcNAc	N-Acetyl-D-glucosamine
HA	Hyaluronic acid/hyaluronan
HCT	Hydroxycinnamoyl-CoA:shikimate/quinate hydroxycinnamoyltransferase
HSQC	Heteronuclear single-quantum coherence
MALDI-TOF MS	Matrix-assisted laser desorption/ionization time-of-flight mass spectrometry
NADPH	Reduced nicotinamide adenine dinucleotide phosphate
NL	Nanolignin
NMR	Nuclear magnetic resonance
PAL	Phenylalanine ammonia-lyase
SEM	Scanning electron microscopy
TEMPO	2,2,6,6-Tetramethylpiperidin-1-oxyl
UDP	Uridine diphosphate
UDP-GlcA	UDP-glucuronic acid
UDP-GlcNAc	UDP-N-acetyl-D-glucosamine
UV	Ultraviolet
